# Profiling the Interrogee: Applying the Person-Centered Approach in Investigative Interviewing Research

**DOI:** 10.3389/fpsyg.2021.722893

**Published:** 2021-11-03

**Authors:** Nicola Palena, Letizia Caso, Lucrezia Cavagnis, Andrea Greco

**Affiliations:** ^1^Department of Human and Social Sciences, University of Bergamo, Bergamo, Italy; ^2^Department of Human Sciences, Libera Università Maria SS. Assunta University, Rome, Italy

**Keywords:** profiling, lie detection, investigative interviewing, person-centered approach, latent profile analysis

## Abstract

In the past, deception detection research has explored whether there were specific personal characteristics that were related to lying and found that factors such as personality and morality are indeed related to lying. However, past research has usually focused on a variable-centered approach. Yet, a person-centered might be more suitable here as it allows for the study of people in an integrative manner. In this experiment, 673 students completed a questionnaire which included measures of the five factors of personality, the level of moral disengagement, the perceived cognitive load when lying, lying strategies, frequency of lying and the LiES scale, a tool measuring the tendency to tell self-serving, altruistic and vindicative lies. We performed a Latent Profile Analysis to integrate personality, moral disengagement, and perceived cognitive load scores into specific profiles. Then, we related profile membership to lying behavior. We obtained four profiles, and found that extraversion, moral disengagement, and the perceived cognitive load contributed most to profile differences. We also found that lying frequency did not differ across profiles, whereas lying tendency did. In conclusion, our results suggest that several facets of the individual play a joint role in lying behavior, and that adopting a person-centered approach might be a good strategy to explore the role of interpersonal differences in lie detection research.

Lying is a common human behavior (DePaulo et al., 1996), and people lie for several reasons (Vrij, 2008): we can lie to gain material advantage, to avoid punishment and loss (DePaulo et al., 1996; Bok, 1999), to avoid harming other people (Vrij, 2008; Levine and Schweitzer, 2015), and, sadly, also to harm others (Guthrie and Kunkel, 2013; Hart et al., 2020).

Although people find lying deplorable (The Global Deception Research Team, 2006), research suggests that lying is frequent (DePaulo et al., 1996; Engels et al., 2006; Lawson and Leck, 2006; Tyler et al., 2006; Vrij, 2008). Several factors can influence deceptive behavior. Men tell more self-oriented lies than women (Rowatt et al., 1999), whereas women tell more other-oriented lies than men (DePaulo and Bell, 1996). A link between the traits of the Five Factor model of personality (Costa and McCrae, 1992) and deception also appears to emerge (Buss, 1992; Kashy and DePaulo, 1996; Wiebe, 2004; Weiss and Feldman, 2006; Vrij, 2008; Williams et al., 2010; Conrads et al., 2013; Eshet et al., 2014; Michikyan et al., 2014; Gylfason et al., 2016). For example, Hart et al. (2020) explored the link between personality and three different types of lies: self-serving lies (lies told to avoid consequences), altruistic lies (lies told to benefit another person), and vindicative lies (lies told to harm another person). They found that lower scores on openness, conscientiousness, extraversion and agreeableness and higher scores on neuroticism correlated with an increased tendency to tell self-serving lies, whereas lower scores on conscientiousness and agreeableness correlated with an increased tendency to tell altruistic and vindicative lies, respectively.

Further, factors other than personality also influence deceptive behavior. Indeed, interpersonal differences have been found to influence both senders and receivers in lie detection tasks (Levine, 2010; Caso et al., 2018, 2019). Morality is one of the factors playing a role in lie production, with people higher in morality lying less than people low in morality. Yet, humans can still adopt “bad behaviors” by distancing themselves from it through moral disengagement, a process lying on eight different mechanism that reframes immoral behaviors as morally acceptable (Bandura, 1986, 1999). Indeed, moral disengagement showed to be positively related with cheating and lying (Barsky, 2011; Šukys, 2013; Tasa and Bell, 2017), as well as with bullying (Gini, 2006; Obermann, 2011), unethical decision making (Detert et al., 2008), organizational transgressions (Bandura et al., 2000; Moore, 2008), and the risk of developing antisocial behavior (Hyde et al., 2010).

The cognitive load experienced when lying is another important factor. It has been suggested that, in general, people experience a higher cognitive load when lying than when telling the truth (Vrij, 2015), a result that has been found also in brain imaging research (Christ et al., 2008). Additionally, research shows that when people perceive a higher cognitive load they might refrain from lying (Mann et al., 2015).

Summarizing, the available literature suggests an association between deceptive behavior and both personality and moral disengagement, but it has not explored how they interact nor how they relate to lying. Yet, such interactions, are important and can be studied via the person-centered approach. According to Magnusson (1998), it allows for the study of individual functioning through an integrative view, in contrast to the traditional variable-centered approach that considers the individual’s characteristics in isolation. Further, the person-centered approach groups individuals through a data-driven process rather than by predetermining criteria (Lewandowski et al., 2014), and accounts for the correlations between the various factors (variables). Hence, it has been suggested that the person-centered approach may predict individuals’ future behavior better than traditional approaches (Magnusson, 1992).

The person-centered approach has already been applied in marketing research (Grieco et al., 2015; de Oña et al., 2016; Del Chiappa et al., 2018), dementia studies (Morganti et al., 2020), educational (Rodríguez et al., 2014; Jaakkola et al., 2015), clinical (Lewandowski et al., 2014; Steca et al., 2016), and investigative psychology (Canter and Youngs, 2009; Sea et al., 2020). Yet, to the best of our knowledge, there is only one available study that applied the person-centered approach in lie detection research.

Fornaciari et al. (2013) evaluated the linguistic features of 31 statements issued in Italian Courts and found that some profiles (which they labeled as “friendly,” “organized,” and “insightful”) were better classified by a machine learning algorithm than others (such as “uncooperative” people). However, this study did not account for moral disengagement, and it did not explore neither the tendency to lie nor the strategies used by people belonging to specific profiles.

## Aims of the Study

The aim of this study was to explore how the five factors of personality (Costa and McCrae, 1992), the level of moral disengagement (Bandura et al., 1996; Caprara et al., 2006), and the perceived cognitive load when lying integrate to form specific profiles and how such profiles: (i) are associated with the tendency to tell three types of lies (altruistic, antisocial, and self-serving); (ii) relate to the use of specific strategies employed to deceive, and; (iii) affect lying frequency.

We did not have clear expectations concerning how many profiles would emerge, what would be the pattern of their scores on personality, moral disengagement, and the perceived cognitive load when lying, and what association the profiles would have with lying strategies. Indeed, (a) to the best of our knowledge, no previous research explored the interaction or relationship between such variables; (b) the correlational pattern of such variables in the whole sample are not very informative when using the person-centered approach, as different profiles are characterized by different correlational pattern, and (c) it is not possible to know such patterns within each profile before the profiles are obtained.

However, research shows that there are both people with a high tendency to lie (sometime referred to as “prolific liars”) and people with low tendency to lie (Serota and Levine, 2015). Further, lying tendency is related dispositional features and personal characteristics (Markowitz and Levine, 2021). Hence, we expected that such conclusions would also show up in our analyses.

To provide an example, the (cor)relation between moral disengagement and extraversion may be different between individuals. Assuming that high moral disengagement and low extraversion are related to higher tendency to lie, those who show this pattern may be more likely to lie than those who score high on both or those who score low on moral disengagement and high on extraversion. Similarly, people with high moral disengagement and high perceived cognitive load when lying may have a lower tendency to lie than people who show high moral disengagement but low perceived cognitive load. Indeed, the first may be morally disengaged when lying, but they could still avoid it when possible if they feel that it is too cognitively demanding for them. On the contrary, those who are again morally disengaged but find lying cognitively simple, may lie more.

Building on the above, we predicted that at least one profile would be marked by a combination of variables associated with high tendency to lie (e.g., low extraversion, high moral disengagement, and low perceived cognitive load) (H1a) and one would be marked by a combination of variables associated with a lower tendency to lie (e.g., low moral disengagement and high perceived cognitive load) (H1b).

Further, we predicted that: (i) the more a profile included characteristics associated with lying, the more frequently participants in that profile would lie (H2); (ii) profiles marked with a combination of variables associated with high tendency to lie would be associated with telling more vindicative and self-serving lies than the other profiles (H3).

Unfortunately, due to the lack of previous research reported above, it is not possible to state more precise predictions, and indeed liberal, rather than strict, hypotheses are common in person-centered approaches (Steca et al., 2016).

## Methods

### Participants

The only inclusion criterion was that the participant must be over 18. In total, 673 participants took part in the study. Age ranged from 18 to 91 years old (*M* = 32.63, *SD* = 15.14). About 73% were female, and about 26% were male. About 57% were students (about 17% of them were working students), about 1% was unemployed, about 5% were retired from their work and about 35% were active workers.

### Variables and Instruments

Personality was evaluated through the Italian revised version (I-TIPI-R, Chiorri et al., 2015), of the original 10-Item Personality Inventory (TIPI, Gosling et al., 2003). The original TIPI is a short and validated measure of the five factors of personality domains (Gosling et al., 2003) and has already been successfully used in deception research (Hart et al., 2020). In the TIPI, the participant answers two items for each of the five personality factors on a scale ranging from 1 (disagree strongly) to 7 (agree strongly) and the answers for the five pairs are combined, yielding to a score ranging from 2 to 14. However, Chiorri et al. (2015) found that Italian version of the TIPI provided by Gosling is inadequate for an Italian sample, and thus created a revised version, the I-TIPI-R. They showed that it has an adequate factor structure, test-retest reliability, self-observer agreement, convergent, and discriminant validity with the Big Five Inventory (Ubbiali et al., 2013). The five factors were calculated by averaging the items composing each factor (Gosling et al., 2003).

Morality was measured via the Moral Disengagement Scale, consisting of 32 items exploring the eight dimensions of moral disengagement (for a detailed overview, see Bandura, 1986, 1999; Bandura et al., 1996; Caprara et al., 2006): *moral justification* (seeing the action as acceptable when at the service of valued social or moral purposes), *euphemistic labeling* (using language to describe/mask the immoral action to make it appear as socially acceptable), *advantageous comparison* (comparing immoral actions to actions that are even worse), *displacement of responsibility* (justifying reprehensible actions as a consequence of social pressure, switching the agency of the action itself from the perpetrator to the community or to other people), *diffusion of responsibility* (the responsibility of the action is not a consequence of the action of the single individual: the perpetrator’s behavior is attributed to the behavior of others), *disregarding the consequences* (distorting or ignoring the consequence of reprehensible behavior adopted to reach one’s goal), *dehumanization* (the victim is divested of human qualities and/or s/he is attributed bestial qualities), and *attribution of blame* (the victim is seen as responsible for the behavior adopted by the perpetrator). The total level of moral disengagement was calculated by averaging the scores of the 32 items.

The participants also reported their perceived cognitive load when lying. The question was “*How much mentally challenging do you find lying?”* The answer ranged from one (not at all) to five (very much). We decided to measure the perceived, rather than the actual, level of cognitive load because we were interested in what the participants experience when lying. Indeed, our prediction is that perceiving lying a cognitively demanding task may act as a deterrent of lying (Mann et al., 2015), which may in turn explain the result whereby one of the most common strategy is to keep the story simple (Vrij, 2008; Leins et al., 2013; Verigin et al., 2019).

For what concern the dependent variables, lying tendency was measured via the LiES scale, consisting of 16 statements which the participants rate from 1 (strongly disagree) to 7 (strongly agree). This tool examines three dimensions concerning lying behavior: self-serving lies, altruistic lies and vindicative/antisocial lies (Hart et al., 2020). Some examples items are “*To avoid embarrassment, I lie*” (self-serving lies), “*I lie in order to make people feel better*” (altruistic lies), and “*I lie for revenge*” (vindicative lies). The scores for each factor are calculated by averaging the answers to the items concerning each specific dimension. Due to the lack of validation of this scale in Italian language, the internal consistency of the three factors was calculated and a confirmatory factor analysis was run.

For what concerns lying strategy, research suggests that people strategize and adopt information management in order to increase the odds of being believed when lying (Verigin et al., 2019). People regulate their social behavior to improve their self-presentation (DePaulo, 1992; DePaulo et al., 2003) and try to behave and speak in order to avoid behaviors associated with lying and to appear truthful (Vrij et al., 2010). This was also showed in Leins et al. (2013), were the results indicated that among the strategies people used when they lie there are the exploitation of previous experiences and keeping a story simple. However, different people may use different strategies. For these reason, we asked our participants to select their preferred strategy among a list derived from Verigin et al. (2019). The options were: *“keeping the statement clear and simple,” “telling a plausible story,” “avoidance,” “embedding the lie,” “providing unverifiable details,” “matching the type of details between lies and truths,” “reporting from previous experiences,” “matching the amount of detail between lies and truths,” “use complete fabrications,” “other strategies.”* Further, following Verigin et al. (2019), participants rated on a scale ranging from 1 (not at all) to 10 (very) how important they perceive verbal and non-verbal strategies to get away with their lies.

In the same way as done in previous research (Serota et al., 2010; Serota and Levine, 2015), participants also reported how many lies of the following types they told in the last 24 h: white lies, exaggerations, omissions, falsifications, and embedded lies.

### Procedure

First, due to the need for a translation, for some items, an Italian researcher with high proficiency in English who was unaware of the aims of the experiment translated the LiES scale and the definition of lying strategies of Verigin et al. (2019) in Italian and then back into English. Two other researchers, again Italian mother-tongue with high proficiency in English, evaluated the quality of the translations of each item on a scale ranging from one to three (one corresponding to “not coherent,” two to “coherent,” and three to “very much coherent”). Inter-rater agreement ranged from 93.75 to 100%.

Then, an online Google Modules survey was shared with university students and on social media. After providing informed consent, the participants read an explanation of their task and then completed the survey. Participation was voluntary, no reward was offered. Filling the questionnaire took about 12 min. The study was conducted in accordance with the Declaration of Helsinki (World Medical Association (WMA), 2004) and with the ethical guidelines for research provided by the Italian Psychological Association (Associazione Italiana di Psicologia, 2015). All data were anonymous.

### Data Analysis Plan

#### Sample Size Calculation

To determine the required sample size, we used GPower (Faul et al., 2007) and set the power at 0.95, α at 0.05, and the effect size *f* at 0.25, as we were interested in at least moderate effect sizes. We run two *a priori* power analyses: one assuming two emerging profiles (needed *N* = 210) and one assuming five (needed *N* = 305; we decided not to consider more than five groups as we aimed to be parsimonious, also to avoid making the interpretation the profiles difficult). However, analyses such as LPA require large sample sizes (>500, see Meyer and Morin, 2016), hence we aimed at reaching a larger sample and stopped when the data collection window closed.

#### Latent Profile Analysis

Profiles have been obtained by a mean of Latent Profile Analysis (LPA), based on participants’ scores on the I-TIPI-R, the Moral Disengagement Scale and on the perceived cognitive load when lying. The aim was to obtain homogeneous groups and to explore whether they differed quantitatively and/or qualitatively. We evaluated five different models, from a one profile solution thorough a five profiles solution. As an estimator, we selected Robust Maximum Likelihood (Yuan and Bentler, 2000; Boduszek et al., 2021). With LPA, there is the risk that untrustworthy solutions are obtained due to local maxima. To reduce such risk, we selected 1,000 random starting values and 250 final stage optimizations. To compare the fits of the different models, we used the Log likelihood, the Akaike Information Criteria (AIC), the Bayesian Information Criteria (BIC), the sample adjusted BIC (SABIC). Lower values indicate better fit. Further, we calculated entropy, which is an indication of how well the profile solution can classify individuals into profiles, with larger values of entropy indicating better classification. Last, we used the Lo-Mendell-Rubin test, which compares a solution with the previous *k*-1 solution. In short, this test examines whether a less parsimonious solution is better than the previous, more parsimonious, solution. A non-significant value indicates that no differences between the two models is present, suggesting that the model with less profiles should be selected.

#### Relating Profiles to Lying

The association between profile membership and the categorial preferred lying strategy was explored by contingency tables analyses.

The association between profile membership and: (i) the tendency to tell altruistic, antisocial, and self-serving lies; (ii) the perceived importance of verbal lying strategies; (iii) the perceived importance of non-verbal lying strategies; and (iv) the frequency of various forms of lying was explored via MANOVAs and subsequent univariate tests. *Post hoc* analyses were conducted with Bonferroni correction. Last, to further illustrate the pros of the variable-centered approach, we conducted a multivariate general linear model with the five factors of personality, the level of moral disengagement, the perceived cognitive load when lying and gender as predictors and the LiES scores as the outcome variables.

## Results

### Preliminary Analyses

Screening of the data showed that, by adopting West et al. (1995) guidelines whereby normality is assumed when asymmetry does not exceed |2| and kurtosis does not exceed |7|, all the seven variables selected to create the profiles were normally distributed. However, none of the variables regarding the frequency of lying was normally distributed (all showed high positive skewness). Hence, they were transformed in their square root (Barbaranelli and D’Olimpio, 2006). The transformation worked for all variables excluded the number of falsifications, which we tried to transform via log10 transformation, but this did not work neither. Hence, this variable was excluded from the analyses.

Since the LiES scale (Hart et al., 2020) is not validated in Italian language, we also tested the validity of this scale. The Cronbach’s alpha was “good” for self-serving (0.83) and altruistic (0.80) lies but “poor” for vindicative lies (0.44). Further exploration showed that item 16 (“*I do not lie in order to intentionally harm people*”) was problematic. When excluding this item, internal consistency raised to 0.81. Hence, we excluded this item from the calculation of the vindicative lies factor. We then conducted a Confirmatory Factor Analysis using the Diagonal Weighted Least Squares (DWLS) estimator, which is considered to be more effective than Maximum Likelihood estimator when dealing with ordered variables (Pastore and Lombardi, 2014; Lionetti et al., 2016). The fit was good [χ^2^(74) = 246.91, *p* < 0.001, CFI = 0.97, TLI = 0.96, RMSEA = 0.059, SRMR = 0.071].

### Latent Profile Analysis

**FIGURE 1:**
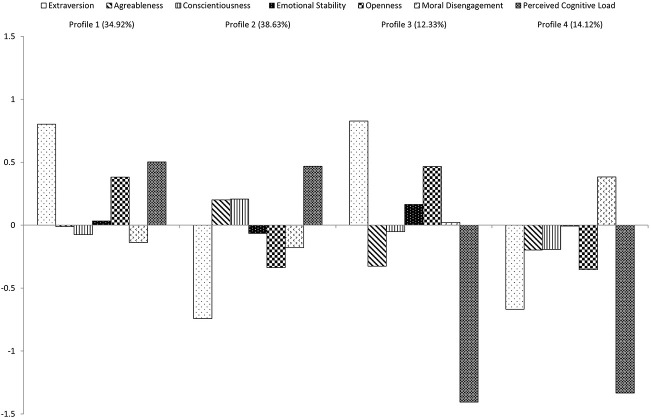
Latent profile analysis plot describing the scores of personality, morality, and perceived cognitive load for each profile. The scores of the six variables which constitute the profiles (personality, moral disengagement, and perceived cognitive load) are reported as Z-scores and their deviation from the grand mean.

Before conducting the LPA, all the selected variables were standardized. The fit obtained for the Latent Profile Analyses are reported in Table 1, whereas Table 2 reports the demographics and the variable scores for each profile. The log likelihood, AIC, BIC, and SABIC were all lower for the five-profile solution. Yet, this solution was untrustworthy as the results may be due to local maxima. Since the issue was not resolved when incrementing the number of the random starts, this solution was ignored. The second-best solution was the four-profile solution, which also showed a significant LMR test. This indicates that the four-profile solution outperforms than the three-profile solution. Entropy of this four profile solution was of .736 which, although not extremely high, can still be acceptable (Muthén, 2021). Hence, we selected this solution. The profiles plot is depicted in Figure 1. Since the profiles are defined with z-scores, each profiles’ z-score on a specific variables indicates the distance between the profile’s mean and the total sample mean (Scholte et al., 2005). Such ranges, although expressed as standard deviations units, can be interpreted as effect sizes in a similar vein of how Cohen’s *d* are interpreted (Steca et al., 2016): small effect (0.2), medium effect (0.5), and large effect (0.8).

**TABLE 1:** Latent profile analysis model fit measures and comparisons.

**Model**	**Log likelihood**	**AIC**	**BIC**	**SABIC**	**Entropy**	**Smallest class%**	**LMR *p***	**LMR meaning**
1	−6569.006	13166.012	13229.177	13184.725	−	−	−	−
2	−6485.864	13015.727	13114.986	13045.134	0.838	26.89	<0.001	2 > 1
3	−6454.105	12968.211	13103.563	13008.311	0.724	13.52	0.098	3 < 2
4	−6422.540	12921.081	13092.527	12971.874	0.736	12.33	<0.05	4 > 3
5	−5938.432	11968.864	12176.404	12030.350	0.917	10.69	<0.05	5 > 4

*n = 673. AIC, Aikake Information Criterion; BIC, Bayesian Information Criterion; SABIC, Sample Adjusted Information Criterion; LMR, Lo, Mendell and Rubin test; LMR meaning = < indicates that the less parsimonious solution is not better than the more parsimonious solution, whereas > indicates the opposite. Solution five is not trustworthy as results may be due to local maxima. The issue was not resolved by incrementing the number of random starts.*

**TABLE 2:** Demographic characteristics of each of the four profiles.

	**Profile 1 [%]**	**Profile 2 [%]**	**Profile 3 [%]**	**Profile 4 [%]**
*N* tot	235 [34.92%]	260 [38.63%]	83 [12.33%]	95 [14.11%]
*N* males	56 [8.32%]	55 [8.17%]	23 [3.42%]	42 [6.24%]
*N* females	178 [26.45%]	202 [30.01%]	59 [8.77%]	52 [7.73%]
*N* other	1 [0.15%]	3 [0.44%]	1 [0.15%]	1 [0.15%]
*M*_*age*_	33.69	33.85	30.23	28.76
*N* student	76 [11.29%]	108 [16.04%]	34 [5.05%]	54 [8.02%]
*N* active worker	146 [21.69%]	124 [18.42%]	46 [6.83%]	37 [5.50%]
*N* unemployed	3 [0.44%]	5 [0.74%]	1 [0.15%]	1 [0.15%]
*N* retired	10 [1.48%]	21 [3.15%]	1 [0.15%]	3 [0.44%]
Extraversion	0.802	−0.742	0.827	−0.668
Agreableness	−0.01	0.201	−0.326	−0.197
Conscientiousness	−0.074	0.208	−0.051	−0.192
Emotional stability	0.034	−0.065	0.165	−0.007
Openness	0.382	−0.337	0.467	−0.353
Moral disengagement	−0.137	−0.178	0.023	0.383
Perceived cognitive load	0.503	0.468	−1.406	−1.334

*The scores of the six variables which constitute the profiles (personality, moral disengagement, and perceived cognitive load) are reported as Z-scores. The percentages are reported respect to the total sample.*

Profile 1 scored high on extraversion, openness, and perceived cognitive load when lying, and slightly lower than the grand mean on moral disengagement (*z* = −0.14). This profile was made up of 235 participants (about 34% of the total sample) and showed a pattern that may be related to low lying tendency. Indeed, high scores on openness (Hart et al., 2020), cognitive load when lying (Mann et al., 2015) and low moral disengagement (Barsky, 2011; Šukys, 2013) were all found to be related to less lying. Profile 1 hence supports H1b.

Profile 2 (260 participants, about 38% of the whole sample), scored low on extraversion and openness, but high on perceived cognitive load when lying. Further, it also scored slightly lower than the grand mean on moral disengagement. Considering that this profile showed both features related to higher lying tendency (lower scores on extraversion and openness, see Hart et al., 2020) and to lower lying tendency (high perceived cognitive load and low moral disengagement), this profile did not support H1.

Profile 3 scored high on extraversion and openness, but low on perceived cognitive load when lying (83 participants, about 12% of the total sample), which partially supported H1a, in particular because of the level of perceived cognitive load.

Profile 4 was made up of 95 participants (about 14% of the total sample), who scored low on extraversion, openness, and perceived cognitive load when lying, but higher than all other profiles on moral disengagement. Since low levels of extraversion, openness, and perceived cognitive load when lying, and high levels of moral disengagement are related lying, this profile support H1a.

To explore the association between gender and profile membership, a chi-square test was run. However, since only six persons selected the option “other,” they were excluded from the analyses. The test was significant, [χ^2^(3) = 20.33, *p* < 0.001]. Less males and more females then expected belonged to profile 2, but more males than females belonged to profile 4.

### Relating Profiles to External Variables

The Profiles obtained with the LPA where related to the dependent variables reported above. There was no significant difference when focusing on the preferred lying strategy, [χ^2^(27) = 29.30, *p* = 0.35].

On the contrary, the importance of verbal and non-verbal strategies when lying showed a significant multivariate difference for both profile membership, Pillai’s trace [*F*_(6, 1318)_ = 3.08, *p* < 0.01], and gender, Pillai’s trace [*F*_(2, 658)_ = 6.35, *p* < 0.01]. The interaction effect was not significant, Pillai’s trace [*F*_(6, 1318)_ = 0.59, *p* = 0.74]. Concerning the univariate effects of profile membership, the difference was significant for the importance attributed to verbal strategies, [*F*_(3, 659)_ = 3.90, *p* < 0.01], partial eta squared = 0.017, but not for non-verbal strategies, [*F*_(3, 659)_ = 0.55, *p* = 0.65], partial eta squared = 0.003. However, none of the pairwise comparisons were significant.

Concerning the univariate effects of sex, the difference was not significant for the importance attributed to verbal strategies, [*F*_(1, 659)_ = 3.71, *p* = 0.054], partial eta squared = 0.006, but significant for non-verbal strategies, [*F*_(1, 659)_ = 12.47, *p* < 0.001], partial eta squared = 0.02. Female participants (*M* = 8.37; *SD* = 1.86) attributed more importance to non-verbal strategies than male participants (*M* = 7.77; *SD* = 2.10).

There was no difference in the frequency of lying, neither for profile membership, Pillai’s trace [*F*_(12, 1974)_ = 1.15, *p* = 0.31], nor for gender, Pillai’s trace [*F*_(4, 656)_ = 1.89, *p* = 0.11] or their interaction, Pillai’s trace [*F*_(12, 1974)_ = 1.04, *p* = 0.41]. Hence, H2 was not supported.

Last, when focusing on the LiES scores, a significant multivariate effect appeared for profile membership, Pillai’s trace [*F*_(9, 1977)_ = 4.28, *p* < 0.001], gender, Pillai’s trace [*F*_(3, 657)_ = 4.42, *p* < 0.01], and their interaction, Pillai’s trace [*F*_(9, 1977)_ = 2.32, *p* < 0.05].

**FIGURE 2:**
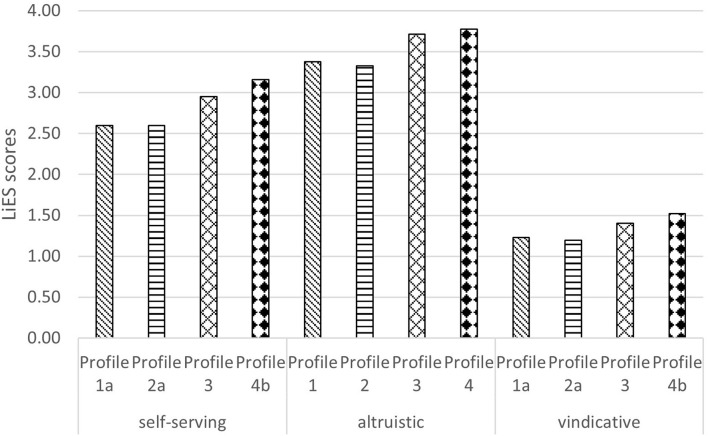
Profiles comparisons of the scores of the three LiES dimensions: self-serving lies, altruistic lies, vindicative lies. Different superscripts indicate a difference with *p* < 0.05. E.g., for self-serving lies Profile 1 and Profile 2 differ from Profile 4 but not from each other nor from Profile 3. The higher the score, the higher the tendency to tell specific types of lies (e.g., self-serving).

Concerning profile membership, a univariate significant effect appeared for self-serving lies, [*F*_(3, 659)_ = 8.36, *p* < 0.001], partial eta squared = 0.040 and vindicative lies, [*F*_(3, 659)_ = 6.75, *p* < 0.001], partial eta squared = 0.030, but not for altruistic lies, [*F*_(3, 659)_ = 2.41, *p* = 0.06], partial eta squared = 0.011. As Figure 2 shows, profile 4 showed a higher tendency to tell self-oriented and vindicative lies than profiles 1 and 2, which did not differ from each other. The effects were medium: For self-serving lies, the difference between Profile 1–4 and Profile 2–4, *g* = 0.52. For vindicative lies, the difference between Profile 1–4 and Profile 2–4, *g* = 0.47. This supports H3.

Concerning gender, a univariate significant effect appeared for vindicative lies, [*F*_(1, 659)_ = 11.04, *p* < 0.01], partial eta squared = 0.020, but not for self-serving, [*F*_(1, 659)_ = 0.09, *p* = 0.76], partial eta squared = 0.00, nor altruistic lies, [*F*_(1, 659)_ = 0.76, *p* = 0.38], partial eta squared = 0.001. Female participants reported a lower tendency to tell vindicative lies (*M* = 1.22; *SD* = 0.61) than male participants (*M* = 1.42; *SD* = 0.82), *g* = −0.30. The Profile by sex interaction was not significant for self-serving, [*F*_(3, 659)_ = 1.06, *p* = 0.36], partial eta squared = 0.005, altruistic, [*F*_(3, 659)_ = 1.77, *p* = 0.15], partial eta squared = 0.008, nor for vindicative lies, [*F*_(3, 659)_ = 2.11, *p* = 0.10], partial eta squared = 0.01.

### Further Analyses

A multivariate general linear model with the five factors of personality, the level of moral disengagement, the perceived cognitive load when lying and gender as predictors and the LiES scores as the outcome variables was conducted for each of the four emerging profiles. The results show that the role of the predictors played a different role within each profile (Table 3). For example, for profile 1, higher levels of moral disengagement and lower levels of perceived cognitive load when lying were associate with a higher tendency to lie. For profile 2, which shows a similar level of perceived cognitive load (*z* = 0.50) to that of Profile 1 (*Z* = 0.47, see Figure 2), cognitive load was no longer a significant predictor of lying tendency. On the contrary, lower level of conscientiousness and high level of moral disengagement were associated with higher tendency to tell self-serving and altruistic lies. For profile 3, low levels of extraversion and of stability are associated with a higher lying tendency, and for profile 4, for example, low levels of conscientiousness and agreeableness are associated with a higher tendency to lie.

**TABLE 3:** Multiple regression with personality, morality, and perceived cognitive load as predictors and LiES scores as the outcome variables for each profile.

			**B**	**Std. Error**	***t***	***p***
Profile 1	Self-serving lies	Intercept	5.064	0.859	5.898	0.000
		Extraversion	−0.019	0.074	−0.253	0.801
		Agreeablessness	0.021	0.059	0.354	0.723
		Conscientiousness	−0.061	0.052	−1.168	0.244
		Emotional stability	−0.124	0.044	−2.787	0.006
		Openness	−0.041	0.058	−0.705	0.481
		Moral disengagement	0.678	0.185	3.667	0.000
		Perceived cognitive load	−0.552	0.130	−4.258	0.000
		Gender	−0.202	0.148	−1.367	0.173
	Altruistic lies	Intercept	6.104	1.247	4.895	0.000
		Extraversion	0.025	0.107	0.230	0.818
		Agreeablessness	0.013	0.085	0.154	0.877
		Conscientiousness	−0.088	0.075	−1.166	0.245
		Emotional stability	−0.054	0.064	−0.833	0.406
		Openness	0.061	0.085	0.721	0.472
		Moral disengagement	0.750	0.269	2.794	0.006
		Perceived cognitive load	−0.840	0.188	−4.461	0.000
		Gender	0.223	0.215	1.038	0.300
	Vindicative lies	Intercept	1.406	0.515	2.730	0.007
		Extraversion	−0.061	0.044	−1.387	0.167
		Agreeablessness	0.019	0.035	0.527	0.599
		Conscientiousness	0.038	0.031	1.227	0.221
		Emotional stability	0.032	0.027	1.207	0.229
		Openness	0.002	0.035	0.063	0.950
		Moral disengagement	0.414	0.111	3.729	0.000
		Perceived cognitive load	−0.212	0.078	−2.725	0.007
		Gender	−0.028	0.089	−0.317	0.751
Profile 2	Self-serving lies	Intercept	3.907	0.826	4.731	0.000
		Extraversion	−0.043	0.068	−0.627	0.531
		Agreeablessness	−0.106	0.059	−1.817	0.070
		Conscientiousness	−0.267	0.055	−4.839	0.000
		Emotional stability	−0.010	0.044	−0.235	0.814
		Openness	0.012	0.057	0.211	0.833
		Moral disengagement	0.877	0.184	4.775	0.000
		Perceived cognitive load	−0.108	0.116	−0.927	0.355
		Gender	−0.163	0.160	−1.020	0.309
	Altruistic lies	Intercept	3.622	1.178	3.076	0.002
		Extraversion	0.057	0.097	0.587	0.558
		Agreeablessness	−0.014	0.083	−0.165	0.869
		Conscientiousness	−0.239	0.079	−3.031	0.003
		Emotional stability	−0.062	0.063	−0.974	0.331
		Openness	−0.012	0.081	−0.149	0.882
		Moral disengagement	0.787	0.262	3.004	0.003
		Perceived cognitive load	0.013	0.166	0.078	0.938
		Gender	−0.225	0.228	−0.985	0.325
	Vindicative lies	Intercept	1.287	0.571	2.254	0.025
		Extraversion	0.107	0.047	2.269	0.024
		Agreeablessness	−0.066	0.040	−1.629	0.104
		Conscientiousness	−0.021	0.038	−0.551	0.582
		Emotional stability	0.001	0.031	0.031	0.976
		Openness	0.033	0.039	0.855	0.393
		Moral disengagement	0.031	0.127	0.244	0.808
		Perceived cognitive load	−0.023	0.080	−0.292	0.770
		Gender	0.114	0.111	1.032	0.303
Profile 3	Self-serving lies	Intercept	5.045	1.360	3.710	0.000
		Extraversion	−0.354	0.134	−2.635	0.010
		Agreeablessness	0.002	0.121	0.016	0.988
		Conscientiousness	−0.205	0.117	−1.748	0.085
		Emotional stability	−0.151	0.099	−1.527	0.131
		Openness	0.107	0.107	0.998	0.322
		Moral disengagement	0.882	0.387	2.282	0.025
		Perceived cognitive load	−0.239	0.200	−1.194	0.236
		Gender	0.197	0.295	0.667	0.507
	Altruistic lies	Intercept	4.991	1.684	2.964	0.004
		Extraversion	−0.023	0.166	−0.135	0.893
		Agreeablessness	0.205	0.150	1.371	0.175
		Conscientiousness	−0.283	0.145	−1.949	0.055
		Emotional stability	−0.046	0.123	−0.371	0.712
		Openness	0.071	0.133	0.532	0.597
		Moral disengagement	0.002	0.479	0.004	0.997
		Perceived cognitive load	−0.278	0.248	−1.122	0.266
		Gender	−0.364	0.365	−0.996	0.323
	Vindicative lies	Intercept	0.809	0.845	0.958	0.341
		Extraversion	−0.148	0.083	−1.773	0.080
		Agreeablessness	−0.047	0.075	−0.629	0.531
		Conscientiousness	0.010	0.073	0.137	0.891
		Emotional stability	−0.180	0.062	−2.922	0.005
		Openness	0.120	0.067	1.799	0.076
		Moral disengagement	0.949	0.240	3.954	0.000
		Perceived cognitive load	−0.013	0.124	−0.101	0.920
		Gender	0.419	0.183	2.288	0.025
Profile 4	Self-serving lies	Intercept	5.136	1.021	5.032	0.000
		Extraversion	−0.282	0.118	−2.384	0.019
		Agreeablessness	−0.221	0.099	−2.238	0.028
		Conscientiousness	−0.241	0.099	−2.444	0.017
		Emotional stability	0.028	0.080	0.352	0.726
		Openness	−0.179	0.093	−1.915	0.059
		Moral disengagement	0.837	0.270	3.097	0.003
		Perceived cognitive load	0.190	0.171	1.107	0.272
		Gender	−0.568	0.240	−2.369	0.020
	Altruistic lies	Intercept	4.087	1.344	3.041	0.003
		Extraversion	−0.435	0.156	−2.795	0.006
		Agreeablessness	−0.113	0.130	−0.865	0.389
		Conscientiousness	−0.313	0.130	−2.414	0.018
		Emotional stability	−0.043	0.105	−0.410	0.683
		Openness	0.095	0.123	0.775	0.441
		Moral disengagement	1.091	0.356	3.063	0.003
		Perceived cognitive load	0.380	0.226	1.682	0.096
		Gender	−0.218	0.316	−0.690	0.492
	Vindicative lies	Intercept	1.365	0.731	1.869	0.065
		Extraversion	−0.023	0.085	−0.268	0.789
		Agreeablessness	−0.219	0.071	−3.101	0.003
		Conscientiousness	−0.083	0.071	−1.174	0.244
		Emotional stability	0.076	0.057	1.328	0.188
		Openness	−0.050	0.067	−0.747	0.457
		Moral disengagement	0.538	0.194	2.778	0.007
		Perceived cognitive load	0.276	0.123	2.253	0.027
		Gender	−0.069	0.172	−0.404	0.687

## Discussion

In this experiment, we aimed at finding subpopulation of participants (Profiles) based on the combination of personality, moral disengagement, and perceived cognitive load when lying. We obtained four profiles, one of which (Profile 4) showed a pattern of scores associated with a higher probability of lying. Indeed, Profile 4 showed low levels of extraversion, agreeableness, openness and perceived cognitive load when lying but high moral disengagement. On the other hand, profile 1 showed a pattern associated with a lower tendency to lie, as it showed high levels of extraversion, openness and cognitive load, but low scores on moral disengagement. The other two profiles showed a somehow mixed pattern, with scores on some variables which, according to the available literature are associated with low lying tendency, and some with a higher one. Our results thus support the idea that people should be seen in an integrative manner, as already suggested previously (Magnusson, 1998), and that focusing on single variables may not describe in detail the specificity and characteristics of each person. According to this approach, people should be seen as a “whole” which is not made as the simple sum of each facet.

When looking at the association between profile membership and lying behavior, we found no differences between profiles for the number of lies told in the previous 24 h. However, our results showed several differences when focusing on the LiES dimensions. We found that participants belonging to profile 4 showed higher tendency to tell self-serving and vindicative lies. This makes sense when read in light of previous research showing that high moral disengagement, low extraversion and low perceived cognitive load when lying are associated with lying (Šukys, 2013; Mann et al., 2015; Hart et al., 2020). Similarly, our result showing that people belonging to profile 1 have a lower tendency to lie fits with previous research, as people belonging to such profile showed, among the others, high openness and perceived cognitive load.

We did not find any association between profile membership and the preferred lying strategy, nor for the importance attributed to verbal nor non-verbal strategies when lying, yet we found an effect for gender. The fact that females attributed more importance to non-verbal behavior than males and showed a lower tendency to tell vindicative lies also fits with previous research (Kashy and DePaulo, 1996).

This experiment also supported the idea that there might be dispositional features that are related with a higher likelihood of lying, but it is important to note that labeling individuals as “liars,” or that some people were born liars while others not, must be avoided: others factors, such as contextual factors, play an important role on lying behavior and tendency (Markowitz and Levine, 2021).

For what concern the practical applications of the present experiment, being this the first study exploring personality profiles in the deception arena, we focused our attention mainly on the exploration and the description of the emerging profiles. Future studies should, however, also focus on the practical application of this approach. For example, profile membership could be used to tailor the best interviewing technique according to each interviewee. Also, once stronger evidence that there are indeed specific profiles related to a higher tendency to tell lies is obtained, such profiles can then be used to select employees for jobs requiring high reliability, such as the intelligence operatives. In this regard, Semrad et al. (2014; 2019; 2020) and Semrad and Scott-Parker (2020) have stressed the importance of defining what personality traits make a good liar, a skill that is particularly important for undercover operatives, and underlined the importance of developing batteries for selection purposes.

Under a theoretical point of view, our study shed light on the importance of studying how several variables play a *joint* role in lying. Personal characteristics and their relationship with lying are no longer studied on their own but, rather, in an integrative manner as if they moderate each other. Here, people are considered as characterized by a constellation of traits (personality), attitudes (moral disengagement), and beliefs/perception (perceived cognitive load when lying) which are differently (cor)related within each emerging profile. For example, we found that people with similar levels of extraversion and openness, but opposite patterns of moral disengagement and cognitive load (see profiles 2 and 4) show different lying tendency. We thus believe that applying the person-centered approach in the investigative interviewing arena might be a useful line of research to reach such goals.

It is important to note, however, that our study had several limitations. First, the effect sizes we obtained were generally small or medium. This could be due to the variables selected to create the profiles. Second, our study was based on self-reported measures. Although relevant research in lying behavior is indeed based on measures such as survey data (Serota et al., 2010; Serota and Levine, 2015) or diary studies (DePaulo et al., 1996; Vrij, 2008), the risk of self-serving bias taking place is at play. Hence future studies should include more objective criteria (e.g., personality rated by observers, objective count of lies told, etc.). Third, our study was cross-sectional, yet it is important to study the effect of personal characteristics longitudinally. In this regard, we also need to point out that we collected data from both students and workers. We think that this should not be an issue. It is indeed difficult to see how personality, which is believed to be stable across life (Costa and McCrae, 1986; Terracciano et al., 2010; McCrae and Costa, 2013), and moral disengagement are related to being a student vs. being a worker, as studying and then moving to the labor market/employment is part of our lives. Yet, future studies should also explore this aspect. Fourth, we assessed the perceived, rather than the actual, level of cognitive load. Although we explained the reasons for this decision above, future research should also explore what role actual cognitive load play. Fifth, future studies should also collect information on geographic location, ethnicity, and socio-economic status.

Despite these limitations, future research should explore in more details what other factors and variables should be used to create such profiles, as well as whether different profiles: (i) are associated with different cues to deception, and (ii) impact on the efficacy of various types of interviewing techniques available to date. In short, a potential practical relevance of this approach would be to focus on different interviewing strategies and/or cues to deception according to the interviewee’s profile membership and to the specific context. Yet, research is still scarce and reaching this aim is likely difficult.

## Data Availability Statement

The raw data supporting the conclusions of this article will be made available by the authors, without undue reservation, to any qualified researcher.

## Ethics Statement

Ethical review and approval was not required for the study on human participants in accordance with the local legislation and institutional requirements. The patients/participants provided their written informed consent to participate in this study.

## Author Contributions

NP and LuC conceived the idea of the study. NP, LeC, AG, and LuC designed the experiment, collected data, conducted the data analysis, interpreted the results, and wrote the manuscript. All authors contributed to the article and approved the submitted version.

## Conflict of Interest

The authors declare that the research was conducted in the absence of any commercial or financial relationships that could be construed as a potential conflict of interest.

## Publisher’s Note

All claims expressed in this article are solely those of the authors and do not necessarily represent those of their affiliated organizations, or those of the publisher, the editors and the reviewers. Any product that may be evaluated in this article, or claim that may be made by its manufacturer, is not guaranteed or endorsed by the publisher.
